# Intratumoral immunotherapy with mRNAs encoding chimeric protein constructs encompassing IL-12, CD137 agonists, and TGF-β antagonists

**DOI:** 10.1016/j.omtn.2023.07.026

**Published:** 2023-07-28

**Authors:** Assunta Cirella, Elixabet Bolaños, Carlos Luri-Rey, Claudia Augusta Di Trani, Irene Olivera, Gabriel Gomis, Javier Glez-Vaz, Beatrice Pinci, Saray Garasa, Sandra Sánchez-Gregorio, Arantza Azpilikueta, Iñaki Eguren-Santamaria, Karmele Valencia, Belén Palencia, Maite Alvarez, Maria C. Ochoa, Álvaro Teijeira, Pedro Berraondo, Ignacio Melero

**Affiliations:** 1 Program of Immunology and Immunotherapy, Cima Universidad de Navarra, 31008 Pamplona, Spain; 2 Navarra Institute for Health Research (IDISNA), 31008 Pamplona, Spain; 3 Program of Solid Tumors, Cima Universidad de Navarra, 31008 Pamplona, Spain; 4 Centro de Investigación Biomédica en Red de Cáncer (CIBERONC), 28029 Madrid, Spain; 5 Department of Immunology and Immunotherapy, Clínica Universidad de Navarra, 31008 Pamplona, Spain; 6 Department of Oncology, Clínica Universidad de Navarra, 28027 Madrid, Spain; 7 Centro Del Cancer de La Universidad de Navarra (CCUN), 31008 Pamplona, Spain; 8 Nuffield Department of Medicine (NDM), University of Oxford, Oxford OX3 7BN, UK

**Keywords:** MT: Delivery Strategies, mRNA, local immunotherapy, IL-12, TGF-β, CD137, 4-1BB

## Abstract

Intratumoral immunotherapy strategies for cancer based on interleukin-12 (IL-12)-encoding cDNA and mRNA are under clinical development in combination with anti-PD-(L)1 monoclonal antibodies. To make the most of these approaches, we have constructed chimeric mRNAs encoding single-chain IL-12 fused to single-chain fragment variable (scFv) antibodies that bind to transforming growth factor β (TGF-β) and CD137 (4-1BB). Several neutralizing TGF-β agents and CD137 agonists are also undergoing early-phase clinical trials. To attain TGF-β and CD137 binding by the constructions, we used bispecific tandem scFv antibodies (taFvs) derived from the specific 1D11 and 1D8 monoclonal antibodies (mAbs), respectively. Transfection of mRNAs encoding the chimeric constructs achieved functional expression of the proteins able to act on their targets. Upon mRNA intratumoral injections in the transplantable mouse cancer models CT26, MC38, and B16OVA, potent therapeutic effects were observed following repeated injections into the tumors. Efficacy was dependent on the number of CD8^+^ T cells able to recognize tumor antigens that infiltrated the malignant tissue. Although the abscopal effects on concomitant uninjected lesions were modest, such distant effects on untreated lesions were markedly increased when combined with systemic PD-1 blockade.

## Graphical abstract

**Figure:**
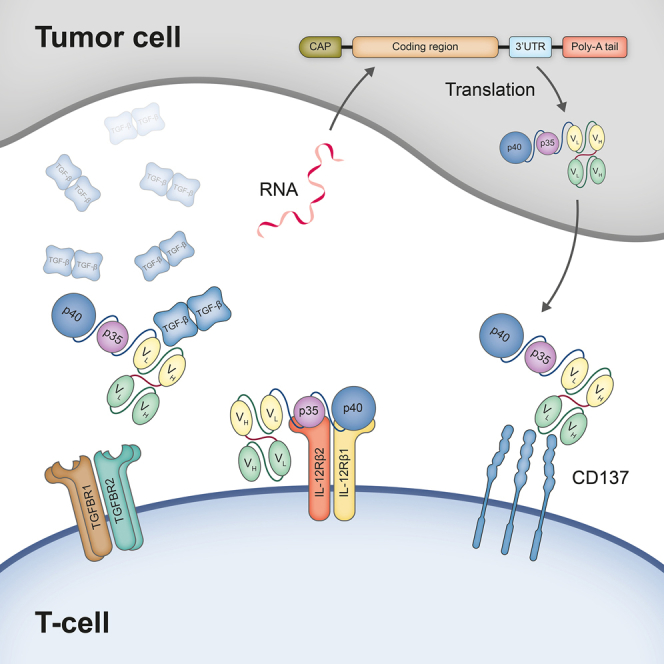

## Teaser

Melero and colleagues designed a chimeric construct encompassing interleukin-12 together with anti-TGF-β and anti-CD137 mAbs, exploiting the mRNA-based technology. Such a chimeric construct was tested for *in vivo* bioactivity and showed antitumoral effects upon intralesional delivery to transplantable mouse tumors. Efficacy was synergistically enhanced upon combination with PD-1 blockade.

## Introduction

Combinations of immunotherapy agents are one of the most avidly pursued routes to improve efficacy against cancer.^1^ In this regard, agents endowed with powerful intrinsic activity often pose serious hurdles in terms of safety. A strategy to mitigate such problems is to locally deliver the immunotherapy agents to malignant lesions in an attempt to maximize therapeutic effects and avoid systemic side effects.^2^ The expectations are that local interventions raise immune responses that would control distant untreated tumors (abscopal or nonenestic effects).^3^ The most widely used immunotherapy agents via intratumoral routes are Toll-like receptor (TLR) agonists, recombinant viruses, and different types of nucleic acids encoding cytokines.^4^ mRNA transfer has several advantages, including rapid dose-dependent expression that, although transient, can be sustained by means of repetition with subsequent doses.^5^ mRNAs encoding immune transgenes have shown efficacy in preclinical models and are being tested in clinical trials.^6^^,^^7^ Naked mRNA dissolved in buffers containing Ca^2+^ could deliver mRNAs to be transiently expressed in the tumor.^8^^,^^9^ However, formulation optimizations are desirable to increase delivery and expression, including nanolipoformulation with ionizable lipid mixtures.^6^^,^^10^ Furthermore, circularization and optimizations of sequences can be implemented for more efficient clinical development. Self-replicating RNA constructs also offer some advantages.^11^

Intratumoral delivery of a number of cytokines is reportedly efficacious against mouse cancer models.^12^ Moreover, a number of these agents are being tested in the clinic. Interleukin-12 (IL-12) is a dimeric cytokine that excels at inducing antitumor immunity but, if used systemically, has a narrow therapeutic window as a result of interferon γ (IFNγ)-mediated toxicity.^13^^,^^14^^,^^15^ Therefore, recombinant versions of IL-12 constructed to be monomeric by a linker (single-chain IL-12 [scIL12])^16^ have been used in multiple gene therapy approaches to locally treat tumor-bearing mice.^11^^,^^17^ These efforts include lipoformulated and naked IL-12-encoding mRNAs.^5^^,^^8^^,^^18^ In the clinic, scIL-12 is showing therapeutic activity upon intratumoral injection when delivered as an IL-12-encoding plasmid DNA or as a lipoformulated mRNA.^19^^,^^20^^,^^21^ The efficacy of scIL-12 mRNA results from its activity on T lymphocytes and natural killer (NK) cells, whereupon it induces activation and IFNγ production, especially if IL-18 is also present.^5^^,^^22^ IL-12 has a key role at inducing Th1 differentiation from Th0 CD4^+^ T cells. In mouse models, the activation of STAT4 by IL-12 was found to be essential for the differentiation of Th1 while inhibiting the differentiation toward a Th2 phenotype.^23^ IL-12 local gene transfer can be combined with systemic immunotherapy agents such as anti-PD-(L)1 monoclonal antibodies (mAbs).^5^^,^^24^ Moreover, various cytokines can be locally delivered together in the form of multiple mixed mRNAs. This approach has been followed with scIL-12, IFNα, granulocyte-macrophage colony-stimulating factor (GM-CSF), IL-15, OX40L, IL-23, IL-36γ, and IL-18.^5^^,^^8^^,^^25^^,^^26^

In addition to cytokines and checkpoint inhibitors, other immunotherapy targets are being preclinically and clinically pursued. Among them, agonist T cell costimulatory mAbs directed to CD137 (4-1BB).^27^ In the case of the current study, bispecific constructs targeting 4-1BB crosslinking to the tumor microenvironment have been tested to avoid dose-limiting liver inflammation as was observed with the agonist antibody urelumab given systemically to patients with cancer.^28^ In preclinical modeling, the anti-CD137 mAb 1D8 is active against various mouse transplantable tumors but also induces a certain degree of liver inflammation in the mice.^29^ Importantly, new CD137 bispecific antibodies targeted to the tumor show evidence for clinical activity.^30^^,^^31^

The tumor microenvironment is enriched in soluble immunosuppressive factors that are known to curtail the functions of the immune cells involved in antitumor responses,^32^ including T and NK lymphocytes, as well as dendritic cells. In that regard, dimeric active TGF-β is reportedly a dominant soluble mediator at downregulating antitumor immunity,^33^^,^^34^ as reviewed by E. Batlle and J. Massagué.^35^ The group of Richard Flavell showed that gene transfer of a dominant-negative variant of TGFBRII to CD8^+^ T cells markedly enhances antitumor immune responses.^36^^,^^37^ Hence, multiple approaches are being followed to neutralize TGF-β activity.^38^ A mAb termed 1D11 that neutralizes all TGF-β isoforms shows antitumor activity in mice.^39^^,^^40^ A humanized version of this mAb is in the clinic (fresolimumab) but has dose-limiting side effects.^41^ Other forms of TGF-β antagonists such as TGF-βR inhibitors and TGF-β traps are also under clinical development via systemic delivery. In the latter case, a fusion protein of TGF-βRII as a decoy receptor was chimerized with the anti-PD-L1 mAb avelumab (bintrafusp alfa) and is currently undergoing clinical development.^42^^,^^43^^,^^44^^,^^45^

In this study, we sought to exploit chimeric constructs encoded by mRNA for intratumoral delivery, seeking synergistic effects of the various functional components in the constructions. Single-chain antibody fragments and scIL-12 were used. A tri-specific construct has been demonstrated to lead to neutralization of TGF-β and immunostimulation via IL-12R and CD137 ligation. Local delivery intends to maximize immunogenicity in a given tumor territory, acting as an *in situ* vaccine.^46^ Therapeutic efficacy results were observed that can be synergized simultaneously using PD-1 blockade.

## Results

### mRNA-encoded trifunctional immunotherapy agents encompassing scIL-12, anti-CD137, and anti-TGF-β

**Figure 1:**
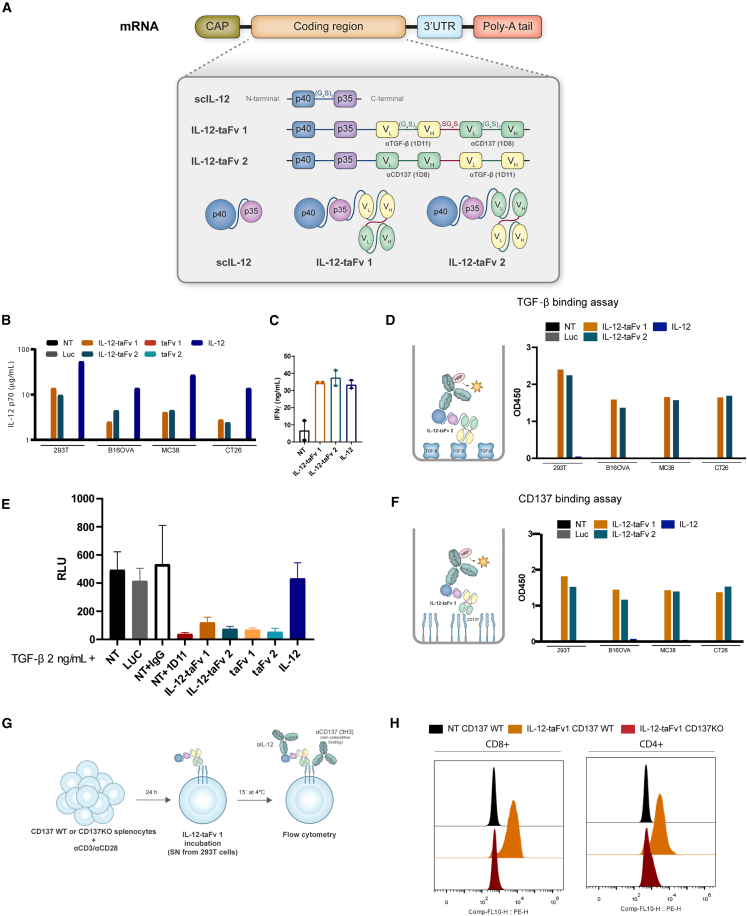
Construction, expression, and function of mRNA-encoded chimeric trifunctional constructs encompassing scIL-12 and scFv anti-TGF-β and anti-CD137 (A) Scheme of the mRNA constructions used that include the incorporated sequences from scIL-12, 1D11 anti-TGF-β mAb, and 1D8 anti-CD137 mAb with the adequate linkers. Representation of the encoded proteins is provided for scIL-12, IL-12-taFv1, and IL-12-taFv2. (B) ELISA-determined concentration of IL-12 in the culture supernatants of the cell lines 293T, B16OVA, MC38, and CT26 transfected with the mRNAs encoding the indicated constructs formulated in TransIT. (C) The indicated supernatants from B16OVA were used to stimulate preactivated spleen T lymphocytes, and IFNγ production in the culture was quantified by ELISA upon 48-h culture. (D) Sandwich ELISA assay as schematized in which recombinant TGF-β1 was coated to the plate surface and, following incubation of the indicated supernatants, was developed by biotinylated anti-IL-12 mAb+streptavidin-HRP. (E) The same supernatants were assayed to neutralize luciferase expression in mink lung epithelial cells carrying a TGF-β-sensitive luciferase reporter system. 1D11 anti-TGF-β mAb was used as a positive control for inhibition. Recombinant TGF-β was added to every condition at 2 ng/mL. (F) Similar sandwich ELISA as in (D) but coating recombinant mouse CD137 to detect binding of the constructs in the culture supernatants of the indicated cell lines transfected with mRNAs encoding the corresponding constructs. (G) Scheme of indirect staining and flow cytometry analysis of CD137 binding to activated mouse T cells expressing CD137. (H) FACS histograms showing the IL-12 coating of activated T cells from WT and *CD137*^−/−^ mice incubated in the presence of the indicated supernatants containing the trispecific constructs. Results are representative of three experimental replicates. In (C) and (E), data are expressed as mean ± SEM. See also Figures S1–S3.

scIL-12 is reportedly active upon intratumoral injection of an mRNA encoding this cytokine.^18^ In order to improve activity, we cloned single-chain fragment variable (scFv) variants of 1D8 and 1D11 mAbs that, respectively, act agonistically on CD137 or antagonize TGF-β.^39^^,^^47^ To make the constructs flexible, spacing linkers were included between the sequence of the synthetic cytokine and the scFv sequences as schematized in Figure 1A,^48^ which represents the mRNA constructs and the predicted proteins. It has been shown that scIL-12 remains functional if present at the N terminus of fusion proteins,^16^ and two versions of the triple constructs were generated with the 1D8 scFv and the 1D11 scFv linked in alternative order (IL-12-tandem Fv1 [taFv1] and IL-12-taFv2) as shown in Figure 1A.

To study the proteins encoded by these *in*-*vitro*-synthesized mRNAs, we transfected several cell lines with the mRNA lipocomplexed using TransIT. Figure 1B shows that in the supernatant of the transiently transfected cells, we could quantify IL-12, denoting the presence of the chimeric proteins. Western blot analyses of the supernatants developed with anti-IL-12 mAb revealed bands of the predicted molecular sizes (Figure S1A). Moreover, the secreted IL-12 was active since the conditioned culture supernatants could induce IFNγ release from mouse splenocytes preactivated for 24 h with anti-CD3 mAb when a 48-h conditioned culture in the presence of the supernatants was carried out (Figure 1C). Next, we studied if the supernatants contained constructs binding TGF-β1 using ELISAs on TGF-β1-coated plates. As shown in Figure 1D, binding of the constructs to recombinant TGF-β1 was substantiated in the analysis of the supernatants of all the transfected mouse cell lines. Moreover, the supernatants successfully inhibited luciferase activity controlled by a TGF-β reporter system based on luciferase as a reporter gene (Figure 1E). TGF-β bioactivity was measured using TGF-β-sensitive mink lung epithelial cells (MLEC) transfected with the reporter system.^49^ In the conditions tested, the supernatants were able to abolish the effects of 2 ng/mL recombinant TGF-β1 to induce luciferase in the reporter system.

Similar ELISA binding experiments were carried out on plastic-bound recombinant mCD137, indicating binding to the target (Figure 1F). Furthermore, binding to CD137 on the surface of activated mouse splenocytes by CD3+CD28 stimulation was observed (Figures 1G and 1H). Flow cytometry staining for extracellular scIL-12 was used to indirectly develop the flow cytometry assay, and activated splenocytes from *CD137*^−/−^ mice were used as a negative control (Figures 1G, 1H, and S1B).

To ascertain if these constructs could be expressed *in vivo*, we used hydrodynamic gene transfer to the liver of mice with the corresponding cDNA constructs in a cytomegalovirus (CMV) promoter-controlled expression plasmid (Figure S2A). 16–18 h following hydrodynamic gene transfer, the serum samples of the mice contained measurable concentrations of IL-12 by ELISA (Figure S2B). Using these sera, it was also possible to demonstrate by ELISA techniques the binding to TGF-β1 that was coated to plastic plates (Figure S2C). The sera with the 1D11 scFv-containing constructs were also able to functionally neutralize TGF-β in the MLEC bioactivity assay (Figure S2D). Binding to recombinant mouse CD137 by the constructs in the serum was also revealed in ELISA assays on mCD137-coated plates (Figure S2E).

The liver can also be gene transferred in a transient manner using lipoplexed mRNA following intravenous administration of the synthetic mRNAs complexed with TransIT reagents. Again, following intravenous injection, readily detectable levels of the constructs could be detected over 6 to 48 h (Figure S3A, left panel), which gave rise to increases in circulating IFNγ (Figure S3A, right panel). Of note, this treatment, given three times to B16OVA-tumor-bearing mice, resulted in some degree of tumor growth control (Figure S3B). However, such mice often succumbed due to systemic toxicity reflected by weight loss and elevated circulating transaminases (Figures S3C–S3E).

Overall, our results show that functional trimeric constructs could be encoded by mRNA and transferred *in vivo* to achieve antitumor immunotherapeutic effects. Given the worrisome safety profile of systemic IL-12,^13^ we sought to investigate the potential of these constructs for intratumoral immunotherapy.^2^

### Therapeutic intratumoral delivery of chimeric mRNAs encoding scIL-12, anti-TGF-β, and anti-CD137

mRNAs encoding the constructs were formulated in Ringer’s lactate and were used to directly inject established subcutaneous B16OVA- and MC38-derived tumors.^5^ Our group has previously demonstrated that the intratumoral administration of IL-12 mRNA in mice bearing subcutaneous tumors is feasible and does not result in observable toxicity.^5^ In B16OVA, substantial concentrations of IL-12 could be measured in the interstitial fluid of tumors injected with the mRNA encoding IL-12 and IL-12-taFv1 (Figure S4A, left panel), and as a result, increased concentrations of IFNγ were also measurable in such recovered tumor interstitial fluid (Figure S4A, right panel). Much lower concentrations of IL-12 were found in peripheral blood (Figure S4B, left panel), and no IFNγ was detected in the circulation (Figure S4B, right panel). This shows the greater safety profile of intratumoral injection. Similar observations were made using mice bearing MC38-derived tumors (Figure S4C).

**Figure 2:**
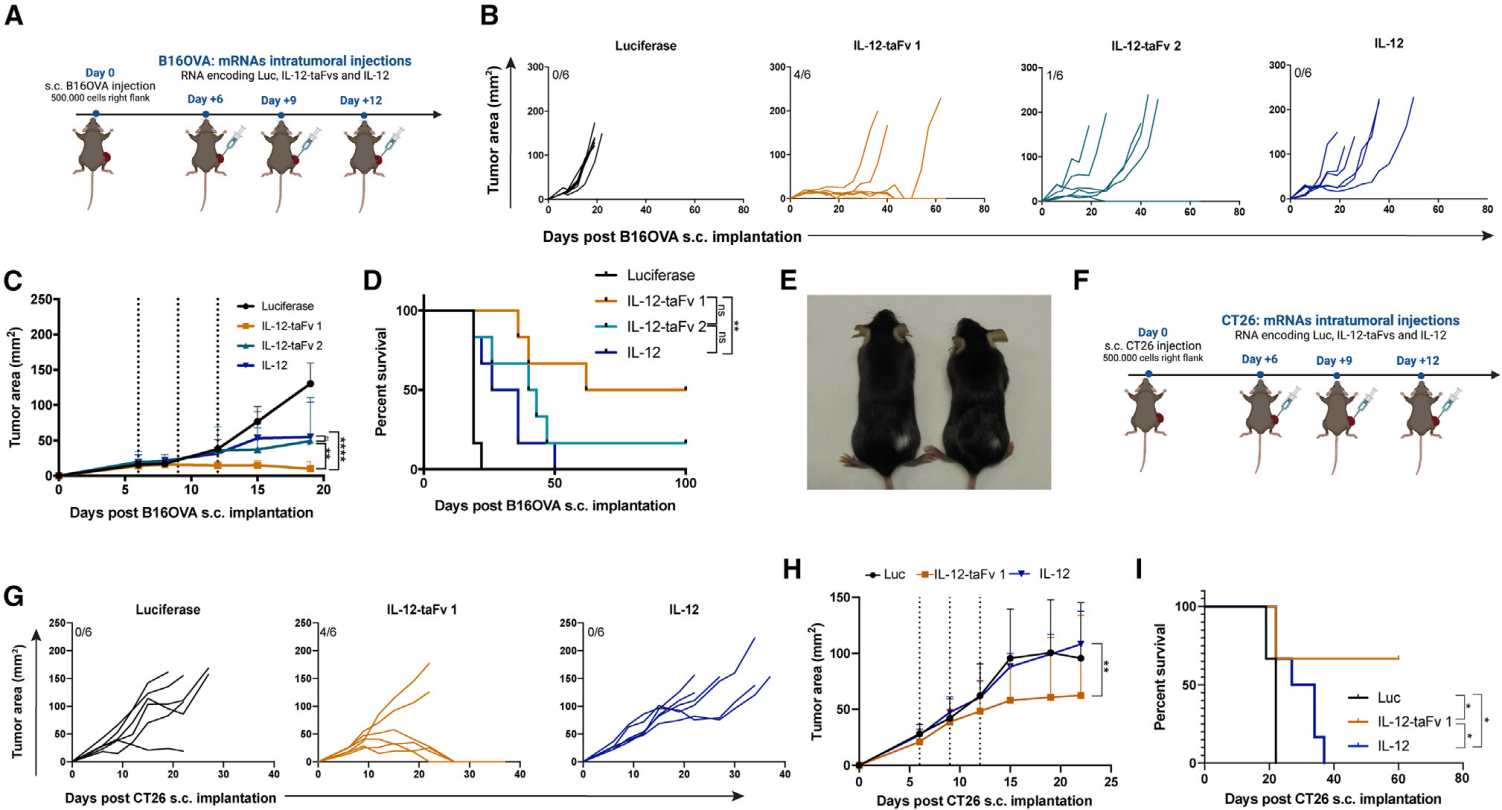
Intratumoral injections of the chimeric mRNA-encoded constructs exert antitumor effects on transplantable mouse tumor models (A) B16OVA tumor cells were engrafted in syngeneic mice to produce established tumors and mRNAs in Ringer’s lactate were injected intratumorally at equimolar doses. (B) Individual follow up of tumor sizes with the fraction of mice achieving complete regressions. (C) Compiled data with statistical comparisons. (D) Overall survival of the mice in the experimental groups. (E) Representative image showing vitiligo in the area where the rejected tumors used to be. (F) Scheme of similar experiments in CT26 tumors engrafted in syngeneic Balb/c mice. (G) Individual tumor follow up in the indicated groups of treatment. (H) Compiled data and statistical comparisons. (I) Overall survival of mice in the indicated treatment groups. Results are representative of two replicates with comparable outcome. In (C) and (H), dotted lines represent the dates of mRNA treatments, and data are expressed as mean ± SD. Longitudinal data were fitted to a third-order polynomial equation and compared with an extra sum-of-squares F test (C and H). Statistical comparisons in (D) and (I) were made using the log-rank test. Statistical significance: ∗p ≤ 0.05, ∗∗p ≤ 0.01, ∗∗∗∗p ≤ 0.0001. See also Figure S4.

Next, we studied the therapeutic effect of repeated intratumoral injections of the mRNA constructs into B16OVA-derived tumors on days 6, 9, and 12 post-tumor cell subcutaneous engraftment (Figure 2A). As shown in Figures 2B and 2C, both IL-12-taFv1 and IL-12-taFv2 exerted antitumor activity. No such activity was seen with control mRNA encoding luciferase, and less efficient activity was observed when scIL-12 was injected at equimolar doses. The advantage in terms of complete regression and survival of IL-12-taFv1 led us to decide to further develop such a construct (Figure 2D). Of interest, successful treatment was associated with vitiligo in the area of the rejected experimental melanomas (Figure 2E). The therapeutic effects of intratumoral IL-12-taFv1 were also observed against CT26-derived tumors implanted in syngeneic Balb/c mice (Figures 2F–2I), in which we also observed complete regression of the injected tumors (4 out of 6), again highlighting the advantage over equimolar doses of mRNA encoding scIL-12 (Figure 2G).

**Figure 3:**
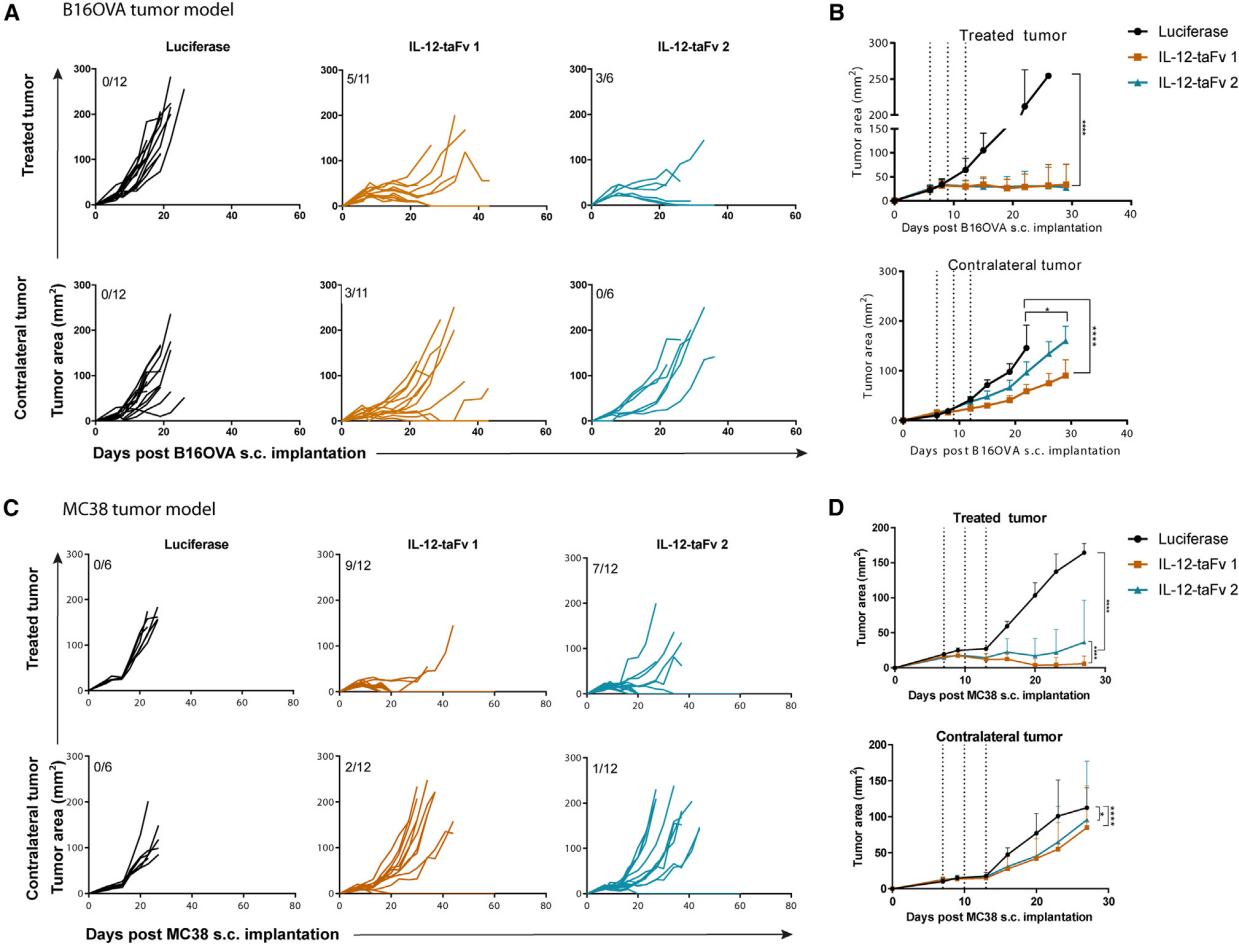
Modest but observable abscopal effects of the mRNA encoding chimeric immunotherapeutic constructs on concomitant untreated tumors In mice bearing bilateral B16OVA-derived tumors for 6 days in opposite flanks, treatment was only given to the right implanted tumor. (A) Individual follow up of tumor size in groups of mice intratumorally treated with the indicated mRNAs. The top panels represent injected tumors and the bottom panels the concomitant noninjected counterparts. (B) Compiled data and statistical comparisons. (C) Similar experiments in mice bearing bilateral MC38-derived tumors for 6 days treated with the indicated mRNA constructs. (D) Compiled data and statistical comparisons. Results are representative of two repetitions with comparable outcomes. In (B) and (D), dotted lines represent the dates of mRNA treatments, and longitudinal data were fitted to a third-order polynomial equation and compared with an extra sum-of-squares F test. Data are expressed as mean ± SD. Statistical significance: ∗p ≤ 0.05, ∗∗∗∗p ≤ 0.0001.

An important next step was to determine if the constructs exerted effects on distant concomitant tumors that did not receive intratumoral treatment. Experiments in Figure 3A show clear effects on the tumors injected with the mRNA constructs in the bilateral B16OVA model that were more favorable in the case of IL-12-taFv1. More importantly, 3 out of 11 complete regressions were observed in the concomitant distant tumors (Figures 3A and 3B). Similar experiments in mice bearing bilateral MC38 tumors also demonstrated the more efficacious bilateral effect of IL-12-taFv1 (Figures 3C and 3D), thus providing another reason to select this construct for subsequent experimental development.

### Efficacy of local immunotherapy with the chimeric mRNA IL-12-taFv1 construct is dependent on the function of CD8^+^ T cells

**Figure 4:**
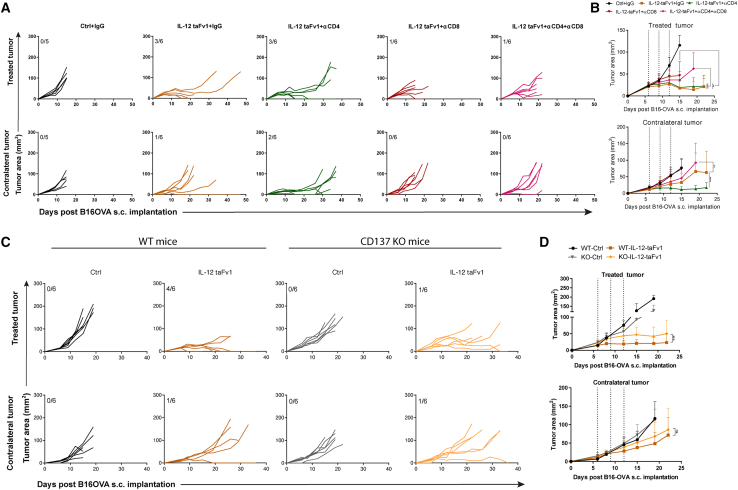
CD8^+^ T cells and CD137 requirements for antitumor activity (A) Selective depletions with anti-CD4 and anti-CD8_β_ monoclonal antibodies of B16OVA bilaterally engrafted mice intratumorally injected with the indicated IL-12-taFv1 mRNA construct on days 6, 9, and 12. Individual sizes of directly treated tumors and concomitant distant tumors are shown. (B) Compilation of data and statistical comparisons. (C) Experiments performed in either WT or *CD137*^−/−^ mice bearing B16OVA tumors as indicated. Tumors were intratumorally treated with Ringer’s lactate (vehicle) or the IL-12-taFv1 mRNA construct and measured over time. (D) Compiled data and statistical comparisons. In (B) and (D), dotted lines represent the dates of mRNA treatments. Results are representative of two repetitions with a comparable outcome. Longitudinal data were fitted to a third-order polynomial equation and compared with an extra sum-of-squares F test (B and D). Data are expressed as mean ± SD. Statistical significance: ∗∗p ≤ 0.01, ∗∗∗p ≤ 0.001, ∗∗∗∗p ≤ 0.0001.

To study the immune requirements underlying the efficacy of our mRNA construct, we performed selective lymphocyte depletion experiments in mice receiving treatments for B16OVA tumors such as those in Figure 3A. Following CD8_β_^+^ T cell depletion or double CD8_β_^+^ T cell and CD4^+^ T cell depletion, the therapeutic effects on the directly treated and distant tumors were almost absent (Figures 4A and 4B). Single depletion of CD4^+^ T cells had no effect and even seemed to enhance efficacy against the contralateral tumor, perhaps as a result of regulatory T cell (Treg) depletion.^50^

Next, we investigated whether CD137 was involved in our therapeutic effects by using *CD137*^−/−^ C57BL/6 recipient mice in comparison to cohoused wild-type control mice. As can be seen in Figure 4C, the therapeutic effect of intratumoral mRNA encoding IL-12-taFv1 was reduced (Figures 4C and 4D).

**Figure 5:**
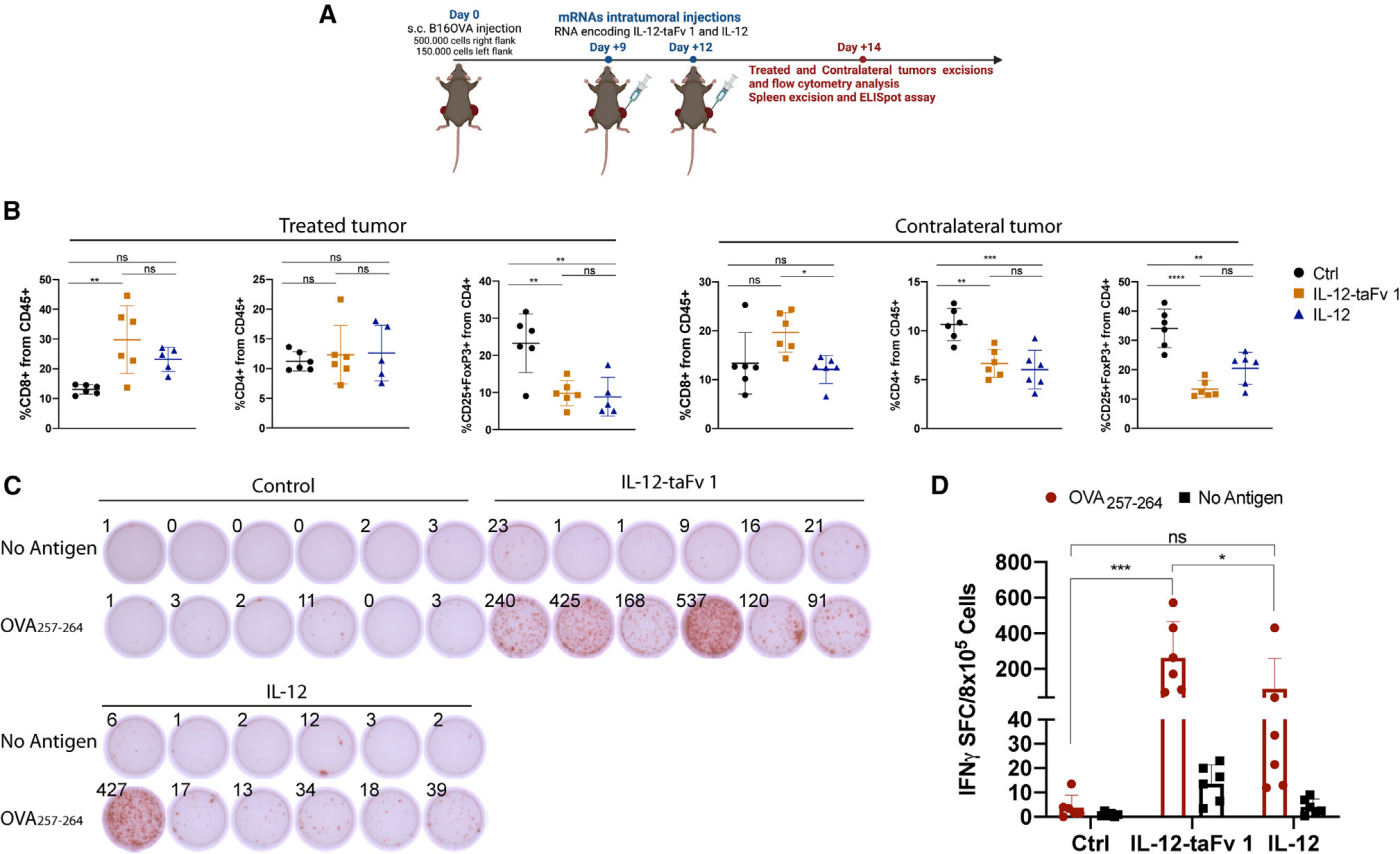
Intratumoral treatment with the mRNA chimeric constructs results in an increase of CD8^+^ T cells both in injected and noninjected lesions and results in specific CD8 systemic immunity (A) Scheme of experiments in mice bearing bilateral B16OVA-derived tumors. (B) Flow cytometry quantification of the percentages of CD8^+^, CD4^+^, and CD4^+^CD25^+^FoxP3^+^ over CD45^+^ leukocytes in the corresponding treated and contralateral tumors following intratumoral injection of the indicated mRNA constructs or vehicle control. (C) Images of IFNγ-ELISpot assays using splenocytes from treated mice as in (A) that were restimulated in a 24-h culture without antigen or with synthetic SIINFEKL peptide. (D) Quantitative data from the ELISpot assays and statistical comparisons. Results are representative of two repetitions with comparable outcomes. In (B), one way ANOVA tests followed by Sidak’s post-test were used for statistical comparisons across groups. In (D), statistical comparisons were made by two-way ANOVA followed by Tukey post-test. Data are expressed as mean ± SD. Statistical significance: ∗p ≤ 0.05, ∗∗p ≤ 0.01, ∗∗∗p ≤ 0.001, ∗∗∗∗p ≤ 0.0001.

In this experimental setting of bilateral B16OVA-bearing mice, we also studied the T cell infiltrates upon treatment as indicated in Figure 5A. A two-dose regimen was implemented in this case in order to prevent the complete eradication of the treated tumors, to permit exams of the tumor microenvironment. As a result of therapy, numbers of CD8^+^ T cells increased in the treated tumors, while Tregs were markedly reduced. Moreover, in the contralateral tumors, a similar trend was observed regarding an increased abundance of infiltrating T cell numbers (Figure 5B).

To establish if CD8^+^ T lymphocytes were systemically recognizing their tumor-associated cognate antigen, IFNγ-enzyme-linked immunosorbent spot (ELISpot) assays were performed using splenocytes from treated mice, as indicated in Figure 5A. Images of IFNγ-ELISpot wells (Figure 5C) and quantitative data (Figure 5D) show that the numbers of CD8^+^ T cells recognizing the canonical ovalbumin (OVA) epitope (SIINFEKL) presented by H-2K^b^ were clearly increased. In this vein, antigen-stimulated OT-I and/or OT-II T lymphocytes exposed in culture to IL-12-taFv1 or IL-12 enhance their production of IFNγ (Figure S5).

As a whole, our data indicate that local immunotherapy injection of the mRNA-encoded trispecific chimeric construct exerts both local and systemic T cell-mediated effects, which are therapeutically beneficial to intratumorally treated mice.

### Intratumoral immunotherapy with the chimeric mRNA IL-12-taFv1 construct synergizes with anti-PD-1 blockade

**Figure 6:**
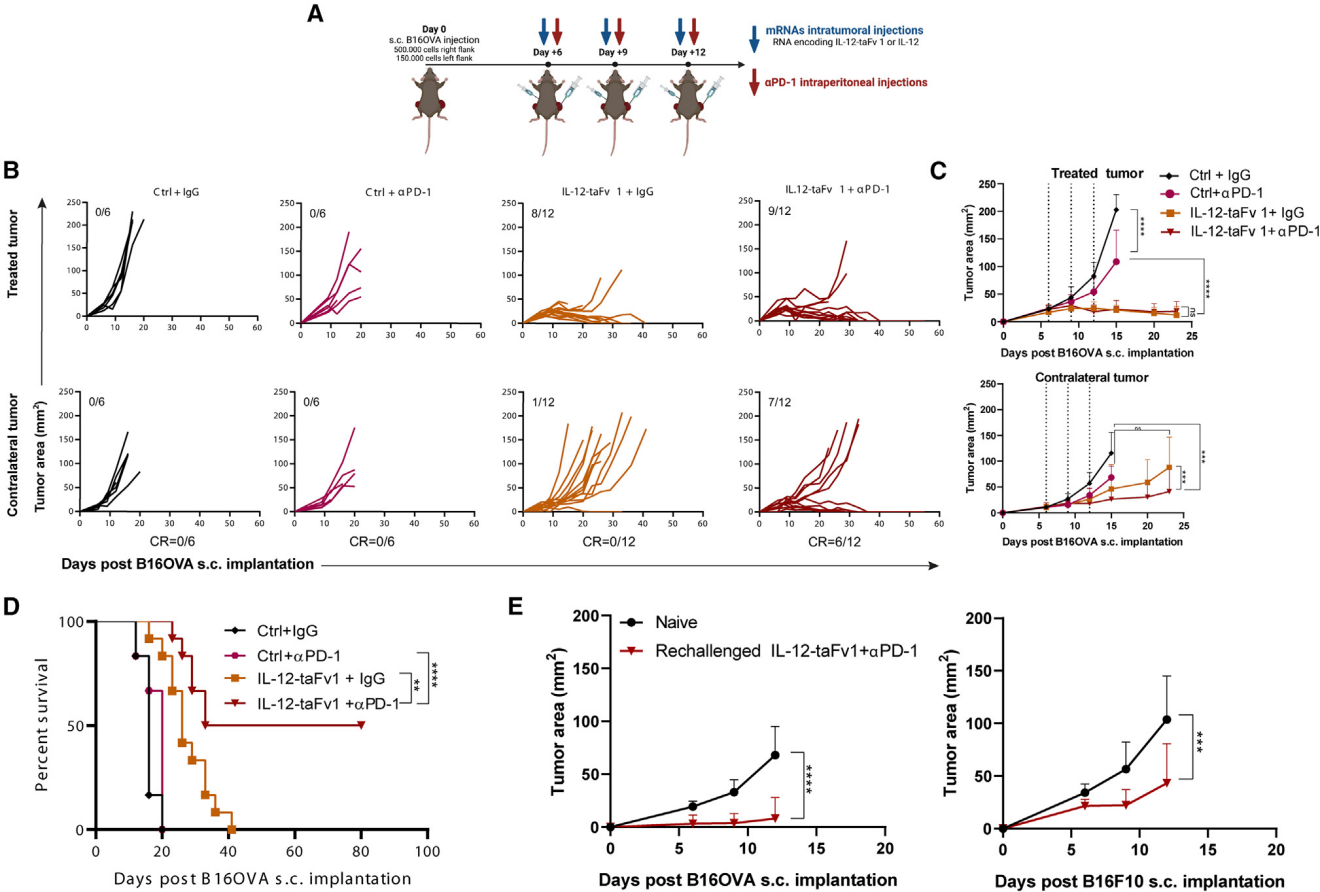
Intratumoral injections of mRNA encoding chimeric constructs encompassing IL-12, anti-TGF-β, and anti-CD137 synergize with systemic PD-1 blockade (A) Schematic representation of the experiments in mice bearing bilateral B16OVA tumors. Mice were intratumorally treated with the IL-12-taFv1-encoding mRNA and systematically with anti-PD-1 mAb as indicated. (B) Individual tumor sizes followed over time of the indicated groups of treatment. The fraction of mice in which complete regression of the tumors was attained is provided for the directly treated and contralateral noninjected tumors. In (C), compiled data and statistical comparisons are provided, and dotted lines represent the dates of mRNA treatments (D) Overall survival of the mice in the indicated groups of treatment. (E) Cured mice from experiments in (B) were rechallenged with tumor cells at least 90 days after being declared tumor free. Mice received B16OVA cells in the right flank and B16F10 cells in the left flank. Tumor sizes were monitored, and naive mice were used as a control. Experiments in (B) and (C) were repeated twice. Data were fitted to a third-order polynomial and compared using an extra sum-of-squares F test (C and E). In (D), statistical comparisons were made using the log rank test. Statistical significance: ∗∗p ≤ 0.01, ∗∗∗p ≤ 0.001, ∗∗∗∗p ≤ 0.0001.

Given that IL-12 is known to increase the expression of PD-L1 on tumor cells via IFNγ,^51^ we sought to investigate whether intratumoral injections of mRNAs encoding IL-12-taFv1 and IL-12 would result in the upregulation of PD-L1 expression on tumor cells. Indeed, intratumoral treatments of IL-12-encoding mRNAs resulted in the increase of PD-L1^+^ CD45^−^ tumor cells (Figure S6). Given the suboptimal systemic effects of the IL-12-taFv1-encoding mRNA construct and taking into account the evidence of PD-L1 overexpression in tumors, we sought to test the combination with anti-PD-1 mAb given systemically (Figure 6A). Results in the B16OVA bilateral model confirmed the efficacy on injected tumors but again showed modest, albeit observable, effects on the distantly implanted tumors (Figure 6B). In this setting, anti-PD-1 mAb given systemically did not achieve any measurable efficacy (Figures 6B and 6C). However, the combination of the intratumoral mRNA IL-12-taFv1 construct with systemic anti-PD-1 mAb resulted in excellent bilateral efficacy, giving rise to complete regression and long-term survival of half of the treated mice (Figures 6A–6D).

Mice surviving long term in these experiments that had remained tumor free for at least 90 days were bilaterally rechallenged with B16OVA in one flank and with B16F10 in the contralateral flank. Naive age-matched mice were used for comparison. As can be seen in Figure 6E, cured mice showed excellent memory to reject B16OVA rechallenge and some degree of cross-reactivity with B16F10, which delayed progression of such tumors. Collectively, our results demonstrate that chimeric immunotherapeutic mRNAs encompassing IL-12, anti-TGF-β, and anti-CD137 can be used for intratumoral approaches, potentiating the effect of checkpoint inhibitors.

## Discussion

In a search for more efficacious alternatives for intratumoral cancer immunotherapies, we tested chimeric mRNAs encoding immunotherapeutic products to be delivered as mRNAs. Based on previous evidence, we sought to combine the effects of IL-12 with TGF-β neutralization and CD137 agonism. The beneficial interaction of such immunotherapy agents using pairs of antibodies and gene transfer has been previously reported.^52^^,^^53^^,^^54^^,^^55^^,^^56^^,^^57^ IL-12 is efficacious upon intratumoral administration of recombinant viruses and IL-12-encoding nucleic acids,^13^ thus providing the rationale to enhance their function with alternative complementary mechanisms. Using mRNA in this setting is advantageous since a more sustained local presence of the protein is achieved, and the resulting translated product has a better chance of locally mediating its immunomodulatory activity. Conceivably, bioavailability of the encoded proteins in tumor-draining lymph nodes is favored. Furthermore, mRNAs are more easily and economically produced than complex recombinant chimeric proteins. mRNA can be used for intratumoral purposes, but liver gene transfer can also be attained using this organ as an endogenous factory of therapeutic proteins.^58^^,^^59^

It can be envisioned that the IL-12-taFv1 construct will simultaneously bind dimeric TGF-β, act on IL-12 receptors, and, as a consequence, be crosslinked to stimulate CD137. Indeed, crosslinking by dimeric TGF-β could be a major advantage of IL-12-taFv1 as a therapeutic agent. Moreover, the coexpression of the targets may result in selective biodistribution of the locally released construct that would be retained in malignant tissues.

The alternative to these chimeric products would have been the use of a mixture of mRNAs. Such an approach has been followed with other mRNAs encoding cytokines and has some advantages. However, it runs up against the unpredictable dominance of some mRNAs over others in terms of expression, less straightforward kinetics, and less simple clinical development due to the need to handle multiple RNA moieties. The chimeric construct also has the advantage of physically linking various counter receptors and potentially giving rise to synthetic and synergistic biology. Indeed, we observe potent effects on injected tumors that are attributable to the mutual potentiation of the effects of each component in the chimeric construct. mRNA as a toolbox allows the delivery of complex chimeric proteins that are otherwise difficult to manufacture and purify.^6^ For instance, in our case, the construction of a human reactive version would be feasible based on known sequences for the scFv of urelumab,^60^ on the interspecies TGF-β cross-reactivity of the 1D11 scFv, and on the available scIL-12 sequences.^18^

A chimeric molecule encompassing a TGF-β trap (TGFBRII) and type I IFN had been previously tested as encoded by mRNA for *in vivo* treatment with a rationale similar to our studies.^61^ Local and regional interference with TGF-β is likely to locally improve dendritic cell functions^62^^,^^63^ and interfere with Treg differentiation^64^ and Treg suppressive functions.^65^^,^^66^ These effects are desirable in addition to the mentioned functional release of CD8 T lymphocytes and NK cells upon TGF-β blockade,^36^^,^^37^^,^^67^ either directly or through modulation of myeloid-derived suppressor cells.^68^ Of important note, TGF-β blockade reportedly synergizes with agonist mAbs to costimulatory members of the TNFR family including OX40^69^ and 4-1BB.^40^^,^^55^

In our experiments, we observe clear antitumor effects that are weak against concomitant tumors that are not directly injected with the mRNA constructs. This is in spite of marked increases in CD8^+^ T cell infiltrates in the mRNA-treated tumors, with a similar trend in the contralateral tumors growing in the group treated with the chimeric molecule. Intriguingly, the percentage of CD4^+^ T cells in such distant noninjected tumors slightly declined, while no changes were observable in the treated tumors. These observations require further investigations focused on lymphocyte migration cues and on the fate of CD4^+^ T cells in the mice receiving treatment. Moreover, the *in situ* vaccination approach is conducive to the production of functional antigen-specific cytotoxic lymphocytes (CTLs) abundantly present in the spleen and detectable by IFNγ-ELISpot. As expected, the antitumor effects as exerted by the mRNA-encoded chimeric molecule were weaker in mice lacking CD137, thus providing evidence for the relevance of CD137 ligation by the chimeric construct.

Given that IL-12 would elicit IFNγ and thereby promote PD-L1 expression, we tested the combination of the intratumoral mRNA constructs and systemic PD-1 blockade.^51^ The synergistic bilateral effect on difficult-to-treat mouse tumor models that are completely resistant to PD-1 monotherapy is truly remarkable. In addition, PD-1 blockade is standard of care for multiple malignancies and easy to combine in early clinical development.^70^ IFNγ is also well known for the induction of surface major histocompatibility complex (MHC) class I expression,^71^^,^^72^^,^^73^ thereby facilitating tumor cell recognition by cognate CD8^+^ T lymphocytes.

Intratumoral immunotherapy faces several obstacles, including logistics and the biological and antigenic heterogenicity of metastatic lesions, but offers the opportunity for tolerable synergistic combinations.^74^ Locally releasing more than one bioactivity in the chimeric constructs aims to produce synergistic effects that can be further potentiated using systemically delivered agents. In our case, we intend to keep TGF-β at bay precisely in the tumor tissue area to which we are providing immunostimulation with IL-12 and anti-CD137 agonists. Our target choice for a 4-1BB agonist rather than other costimulatory members of the TNFR family was based on evidence for clinical activity^30^^,^^75^^,^^76^ in contrast to reported failures using anti-OX40 mAbs.^77^^,^^78^^,^^79^ However, other options are possible including engineering CD28, ICOS (inducible T cell costimulator), or CD27 costimulation.

We used naked mRNA for the proof of concept, but to treat larger tumors in humans, we are investigating lipoformulations in order to maximize delivery and expression. This sort of chimeric construct could also be considered to be launched from oncolytic RNA viral vectors or self-replicating RNAs (srRNA).^80^^,^^81^ srRNAs have advantages since small doses may result in larger quantities of transgene expression, as recently demonstrated for IL-12 with a Semliki Forest virus-based srRNA.^82^

Further improvements in mRNA are currently being investigated to optimize the expression of these relatively long messengers.^10^ Transient systemic leakage because of liver expression could be advantageous but also dangerous. To mitigate this potential issue, microRNA (miR) targets can be incorporated into the mRNAs to prevent hepatic expression.^18^^,^^83^ Once the proof of concept for these immunocytokines launched from mRNA is made, other costimulatory mAbs (i.e., anti-OX40, ICOS, CD27) can be considered as well as other cytokines in similar mRNA-encoded formats.

All things considered, our work provides evidence that chimeric constructs encompassing cytokines and two mAb fragments can be locally and systemically delivered by the synthetic mRNA moieties that encode for them. The strategy holds promise for clinical translation improving the effects of existing intratumoral mRNA-based immunotherapies and showing synergistic effects with systemic delivery of checkpoint inhibitors.

## Materials and methods

### Mice

Mice were housed at the animal facility of the Center for Applied Medical Research (CIMA, Pamplona, Spain). Six-week-old female C57BL/6 and Balb/c mice were purchased from Envigo (Barcelona, Spain). *CD137*^−/−^ (B6.Cg-Tnfrsf9^tm1Byk^), OT-I (C57BL/6-Tg(TcraTcrb)1100Mjb/J), and OT-II (B6.Cg-Tg(TcraTcrb)425Cbn/J) mice were bred in our animal facility (CIMA, Pamplona, Spain). All animal experiments were approved by the institutional ethics committee and by the regional government of Navarra (studies 039-21, 097-21, 079-20, 087-21).

### Cell lines and tumor mouse models

HEK293T (293T) and CT26 cells were procured from the ATCC. MC38 cells were kindly provided by Dr. Karl E. Hellström (University of Washington, Seattle, WA, USA) in September 1998.^84^ B16OVA cells were a kind gift from Dr. Lieping Chen (Yale University, New Haven, CT, USA) in November 2001.^84^ The MLEC cell line was provided by Dr. Fernando Pastor (CIMA, Universidad de Navarra, Pamplona, Spain).^49^ Cell lines were cultured in RPMI 1640 medium (Gibco) supplemented with 10% FBS (Sigma-Aldrich), 100 U/mL penicillin, 100 μg/mL streptomycin (Gibco), and 5 × 10^−5^ mol/L 2-mercaptoethanol (Gibco). HEK293T and MLEC cells were maintained with DMEM high glucose (Gibco) supplemented with 10% FBS, 100 U/mL penicillin, and 100 μg/mL streptomycin. B16OVA and MLECs cells were supplemented with 400 and 250 μg/mL geneticin (Gibco), respectively. All cell lines were grown in a humidified incubator with 5% CO_2_ at 37°C for at least 7 days before inoculation into mice. All cell lines were routinely tested for mycoplasma contamination using the MycoAlert Mycoplasma Detection Kit (Lonza).

For the B16OVA, MC38, and CT26 unilateral tumor model, C57BL/6 mice were subcutaneously injected with 5 × 10^5^ tumor cells in the right flank. For the B16OVA and MC38 bilateral tumor model, C57BL/6 mice were subcutaneously injected with 5 × 10^5^ tumor cells in the right flank and 1.5 × 10^5^ cells in the left flank on day 0. For tumor rechallenge, mice that were B16OVA tumor free at least 90 days after treatment received a subcutaneous injection of 5 × 10^5^ B16OVA and B16F10 cells in the right and left flanks, respectively, in a total volume of 50 μL PBS.

### Plasmids, mRNA synthesis, and *ex vivo* transfection

Variable heavy (V_H_) and variable light (V_L_) sequences for the anti-TGF-β and anti-CD137 were obtained by sequencing of each hybridoma. The variable domains of the 1D11 or 1D8 fragments were fused via a (G_4_S)_5_ linker, and the two scFvs of 1D11 and 1D8 were fused via a SG_4_S linker.^48^ scIL-12 was fused to the taFvs antibodies via a (G_4_S)_3_ linker.^16^^,^^85^ The protein sequences of IL-12-taFvs were designed as following starting from N terminus IL-12-taFv1: IL-12p40-(G_4_S)_3-_IL-12p35-(G_4_S)_3_-1D11_VL_-(G_4_S)_5_-1D11_VH_-SG_4_S-1D8_VL_-(G_4_S)_5_-1D8_VH_, IL-12-taFv2: IL-12p40-(G_4_S)_3-_IL-12p35-(G_4_S)_3_-1D8_VL_-(G_4_S)_5_-1D8_VH_-SG_4_S-1D11_VL_-(G_4_S)_5_-1D11_VH_. Protein sequences of taFv1 and taFv2 used as controls were the same as the IL-12-taFvs but were devoid of scIL-12.

The cDNA sequences encoding mRNAs were cloned by GeneScript in the pUC57-Kan vector containing a T7 promoter upstream of the cDNAs and followed by 2 tandem repetitions of the 3′ UTR sequence of the human β2-globin cDNA and a 60 poly A tail. The cDNA sequence encoding construct DNAs were cloned by GeneScript in the pcDNA3.1(+) vector.

The mRNA production was performed as previously described.^5^ For some experiments, the mRNAs were formulated with TransIT-mRNA Transfection Kit (Mirus Bio) for cell transfections, according to the manufacturer's instructions.

### MLEC luciferase assay

For the luciferase assay, 4 × 10^4^ MLEC cells were plated in a 96-well plate and allowed to attach for 5 h in a humidified incubator with 5% CO_2_ at 37°C. Supernatants from mRNA-transfected cells or serum from hydrodynamically injected mice were incubated with rhTGF-β1 (Peprotech cat. 100-21) at 2 ng/mL for 30 min at 37°C. Such samples with rhTGF-β1 were added to MLEC cells and then incubated in a humidified incubator with 5% CO_2_ at 37°C for 1 h. Cells were then washed with PBS and incubated overnight in 0.1% BSA DMEM. After 16–18 h, the luciferase assay was performed using Bio-Glo Reagent (Promega, cat. G7940) according to the manufacturer’s instructions. Firefly luciferase signal was detected in an Orion L Microplate Luminometer (Berthold Detection System).

### Western blot assay

For direct protein visualization, supernatants from mRNA-transfected cells were separated on 8% acrylamide SDS-PAGE gel and transferred with Trans-Blot Turbo Transfer System to a PVDF blot membrane. IL-12 protein was detected using an anti-mIL-12 antibody (AP-MAB0853).

### *In vivo* transient mRNA and DNA gene transfer

mRNAs were administered by intravenous or intratumoral injections. Given the different molecular weights of the mRNAs and to ensure equimolar administration, the same amount of mRNA moles (8.7 μmol) was injected for each treatment.

For the intravenous administration in B16OVA tumor-bearing mice, mRNA was formulated in TransIT-mRNA transfection kit (Mirus Bio) reagent and intravenously injected into mice, as previously reported.^48^ For intratumoral administration, mRNAs were formulated in Ringer’s lactate (Grifols) and then injected into the tumor in a total volume of 50 μL.

Hydrodynamic tail-vein injections were performed administrating 10 μg of the indicated cDNA plasmids diluted in 2 mL sodium chloride 0.9% at room temperature in less than 8 s.

### Design of mRNA *in vivo* treatment experiments

To determine IL-12 or IFNγ concentrations in tumor extracts and blood, mRNAs were intravenously or intratumorally injected. To quantify IL-12 and IFNγ tumoral protein concentrations, tumors were excised 6 h following the intratumoral mRNA injections and homogenized using VWR Disposable Pellet Mixers in PBS containing cOmplete, Mini, EDTA-free Protease Inhibitor Cocktail (Roche). For quantification in blood, 100–150 μL of peripheral blood were collected in 50 μL of Heparin (Hospira).

To evaluate the toxicity of systemically injected mRNAs, mice bearing B16OVA subcutaneous tumors were intravenously injected on days +6, +9, and +12 after tumor engraftment. Biochemical analysis was performed on day +13 on mice plasma, and body weights were recorded every day.

To evaluate the therapeutic efficacy of mRNA-encoded proteins, tumor-bearing mice were intratumorally injected with mRNAs formulated in Ringer’s lactate on days +6, +9, and +12 after tumor cell subcutaneous implantation. Tumor sizes were measured twice a week.

To investigate the effects of mRNA treatments on tumor T-cell infiltrate, mice bearing B16OVA tumors were intratumorally injected with the indicated mRNAs diluted in Ringer’s lactate on days +9 and +12 after tumor cell subcutaneous implantation. On day +14, mice were euthanized, and primary and contralateral untreated tumors and spleens were excised, and the cell suspensions obtained were analyzed by flow cytometry and the ELISpot assay.

To evaluate PD-L1 expression in mRNA-treated tumors, mice bearing B16OVA tumors were intratumorally injected on days +8 and +9 with 26.1 μmol saline-formulated mRNAs. 24 h following the last injection, tumors were excised and cell suspensions analyzed by flow cytometry.

For combination studies with anti-PD-1 mAb, 200 μg control immunoglobulin G (IgG) antibody (BE0094) or anti-PD-1 mAb (RMP1-14) were intraperitoneally administered simultaneously with intratumoral Ringer’s lactate formulated mRNAs.

For immune cell depletion studies, 100 μg control (IgG), anti-CD8_β_ (53-5.8), anti-CD4 (GK1.5), or a combination of anti-CD8_β_ and anti-CD4 antibodies were intraperitoneally administered 1 day before the first mRNA intratumoral treatment, twice a week for the next 2 weeks, and once a week thereafter until the end of the experiment. Efficient depletion of CD4^+^, CD8^+^, or both populations was checked on day +14 in peripheral blood samples by flow cytometry.

All the antibodies used for *in vivo* studies were purchased from BioXcell.

### ELISA determinations of cytokine concentrations and ELISpot assays

IL-12p70 protein levels in cell supernatants, supernatants of minced tumor tissue, and serum samples were quantified using commercially available ELISA kits (BD OptEIA Mouse IL-12 (p70) ELISA Set, cat. 555256), according to the manufacturer’s instructions.

For IFNγ determination following splenocyte stimulation with supernatants, splenocytes from wild-type (WT) mice were preactivated with plate-bound anti-CD3 (1 μg/mL) for 24 h and exposed to B16OVA transfection medium. Supernatants were collected after 48 h of incubation and analyzed by ELISA (BD OptEIA Mouse IFNγ ELISA Set, cat. 555138) according to the manufacturer’s instructions. The same ELISA kit was used to detect IFNγ in supernatants of minced tumor tissue and mouse serum samples.

To determine the binding of IL-12-taFvs to their targets, Maxisorp ELISA plates were coated at 4°C overnight with 100 ng m41BB (SinoBiological cat. 50811-M08H) or hTGFβ1 (Peprotech cat. 100-21) recombinant protein diluted in PBS. After plate blocking with PBS 10% FBS, supernatants from the indicated transfected cell lines were incubated for 2 h at room temperature (RT) and then revealed with a biotinylated anti-IL-12 antibody+streptavidin-HRP (BD OptEIA cat. 51–9002812).

The OVA-specific CD8^+^ T cell response was assessed *ex vivo* using a mouse IFNγ-ELISpot Assay kit (BD 551083). Splenocytes depleted of erythrocytes were added to the wells (8 × 10^5^ cells) and then stimulated with synthetic OVA_257-264_ peptide (1 μg/mL) for 16–24 h. IFNγ-producing cells were assessed by counting the spots with reference to input cells according to the manufacturer's instructions.

### Flow cytometry

For tumor microenvironment fluorescence-activated cell sorting (FACS) analysis, tumor samples were collected and incubated in collagenase D/DNase I for 15 min at 37°C. All the specimens were then mechanically disaggregated and filtered through a 70-μm cell strainer to obtain single-cell suspensions. Cell surface was stained with the following fluorochrome-labeled antibodies purchased from BioLegend, anti-CD45-PeCy7 (30-F11) and anti-CD25-BV421 (PC61), and the following fluorochrome-labeled antibodies purchased from BD, anti-CD4-BUV496 (GK1.5) and anti-CD8-BUV395 (53-6.7). For intracellular staining, cells were permeabilized after surface staining with True-Nuclear (BioLegend) following the manufacturer’s instructions and stained with anti-FoxP3-Prcp5.5 (FJK-16S, Invitrogen). Promofluor (Promocell) was used to exclude cell death by gating. Samples were acquired on a CytoFlex LX system (Beckman Coulter).

For the detection of the constructs binding to CD137 on activated T cells, spleens from C57BL/6 WT and *CD137*^−/−^ mice were processed to obtain single-cell splenocyte suspensions as previously described.^50^ Splenocytes were preactivated during 24 h using plate-bound anti-CD3 (17A2, 1 μg/mL) and soluble anti-CD28 (37.51, 2 μg/mL). 2 × 10^5^ splenocytes were incubated for 15 min at 4°C with the indicated conditioned medium from mRNA-transfected 293T cells. Then, the cell surface was stained with the following fluorochrome-labeled antibodies purchased from BioLegend: anti-CD4-BV421 (GK1.5), anti-CD8-BV510 (53-6.7), and anti-IL-12p40-PE (C15.6). As 3H3 (BE0239) antibody does not compete with 1D8 for CD137 binding, 3H3 conjugated to AF-647 dye using a Fluorescent Protein Labeling Kit (Invitrogen, ref. A20173) was used for CD137 staining. Zombie NiR (BioLegend) was used to exclude cell death. Samples were acquired on a CytoFlex S system (Beckman Coulter).

For the analysis of lymphocytes derived from OT-I and OT-II mice, spleens were processed in single-cell suspensions and exposed to supernatant of 293T cells enriched for the indicated chimeric proteins and synthetic OVA peptides, OVA_257-264_ (InvivoGen) for OTI and OVA_323-339_ (NeoMPS) for OT-II-derived lymphocytes. OT-I- and OT-II-derived lymphocytes were maintained in culture separately or in a 1:1 mixture over 48 h. Cells were treated with BD GolgiPlug over 4 h. For cytometry assay, cells were stained with the following fluorochrome-labeled antibodies purchased from BioLegend: anti-CD45-BV510 (30-F11), anti-CD4-BV421 (GK1.5), and anti-CD8-Pe/Dazzle594 (53-6.7). For intracellular staining, cells were permeabilized after surface staining with True-Nuclear (BioLegend) following the manufacturer’s instructions and were stained with anti-granzyme B-FITC (NGZB) (eBioscience), anti-IFNγ-APC (XMG1.2) (BioLegend), and anti-IL-4-PerCP/cyanine 5.5 (11B11) (BioLegend). Zombie NiR (Biologened) was used to exclude cell death. Samples were acquired on a CytoFlex S system (Beckman Coulter).

For PD-L1 analysis on tumor cells, tumors were processed as previously described. Single-cell suspensions were stained with the following fluorochrome-labeled antibodies purchased from BioLegend: anti-CD45-BV510 (30-F11) and anti-PD-L1-PerCpCy5.5 (10F.9G2). Zombie NiR (BioLegend) was used to exclude cell death. Samples were acquired on a CytoFlex S system (Beckman Coulter).

All the samples were treated with FcR-Block (anti-CD16/32 clone 93; BD Biosciences) before the cytometry staining.

### Statistical methods

Flow cytometry analyses were performed with CytExpert software. Means and standard deviations of the mean are presented as averages and error bars unless otherwise indicated in the figure legends. GraphPad Prism v.8 (La Jolla, CA, USA) was used for statistical analysis as indicated in figure legends. When differences are statistically significant, the significance is represented with asterisks according to the following values: ∗p < 0.05, ∗∗p < 0.01, ∗∗∗p < 0.001, and ∗∗∗∗p < 0.0001.

## Data and code availability

Data are available upon reasonable request to the corresponding author.

## Supplemental information

Document S1. Figures S1–S6

Document S2. Article plus supplemental information
