# Supplementary material for: Intratumoral immunotherapy with mRNAs encoding chimeric protein constructs encompassing IL-12, CD137 agonists, and TGF-β antagonists
Source: Mol Ther Nucleic Acids. 2023 Jul 28;33:668–82. doi: 10.1016/j.omtn.2023.07.026 (PMC10462790; doi:10.1016/j.omtn.2023.07.026)
Supplement: Document S1. Figures S1–S6 [file mmc1.pdf]

OMTN, Volume *33*

Supplemental information

Intratumoral immunotherapy with mRNAs encoding chimeric protein constructs encompassing IL-12, CD137 agonists, and TGF-β antagonists

Assunta Cirella, Elixabet Bolaños, Carlos Luri-Rey, Claudia Augusta Di Trani, Irene Olivera, Gabriel Gomis, Javier Glez-Vaz, Beatrice Pinci, Saray Garasa, Sandra Sánchez-Gregorio, Arantza Azpilikueta, Iñaki Eguren-Santamaria, Karmele Valencia, Belén Palencia, Maite Alvarez, Maria C. Ochoa, Álvaro Teijeira, Pedro Berraondo, and Ignacio Melero

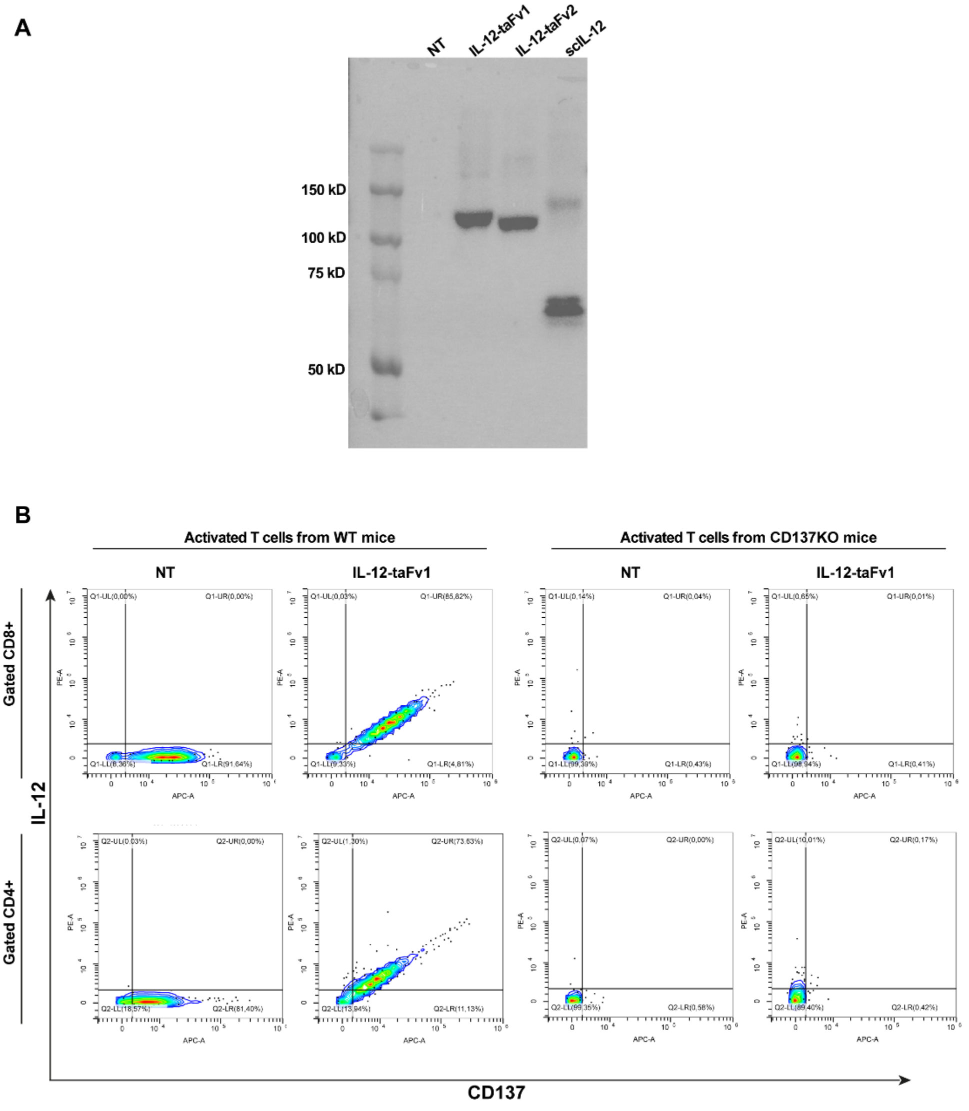

Figure S1

Figure S1. Western blot analyses of the supernatants and dot plots corresponding to experiments in Figure 1G.

(A) Supernatants of 293T cells transiently transferred with the indicated lipoplexed mRNAs were developed by immunoblot with anti-IL-12 mAb. Molecular weight markers are provided. (B) FACS dot plots from figure 1G of the double staining for CD137 (using an antibody that does not compete with 1D8) and IL-12. T cells were electronically gated for CD4 and CD8. Representative data are shown.

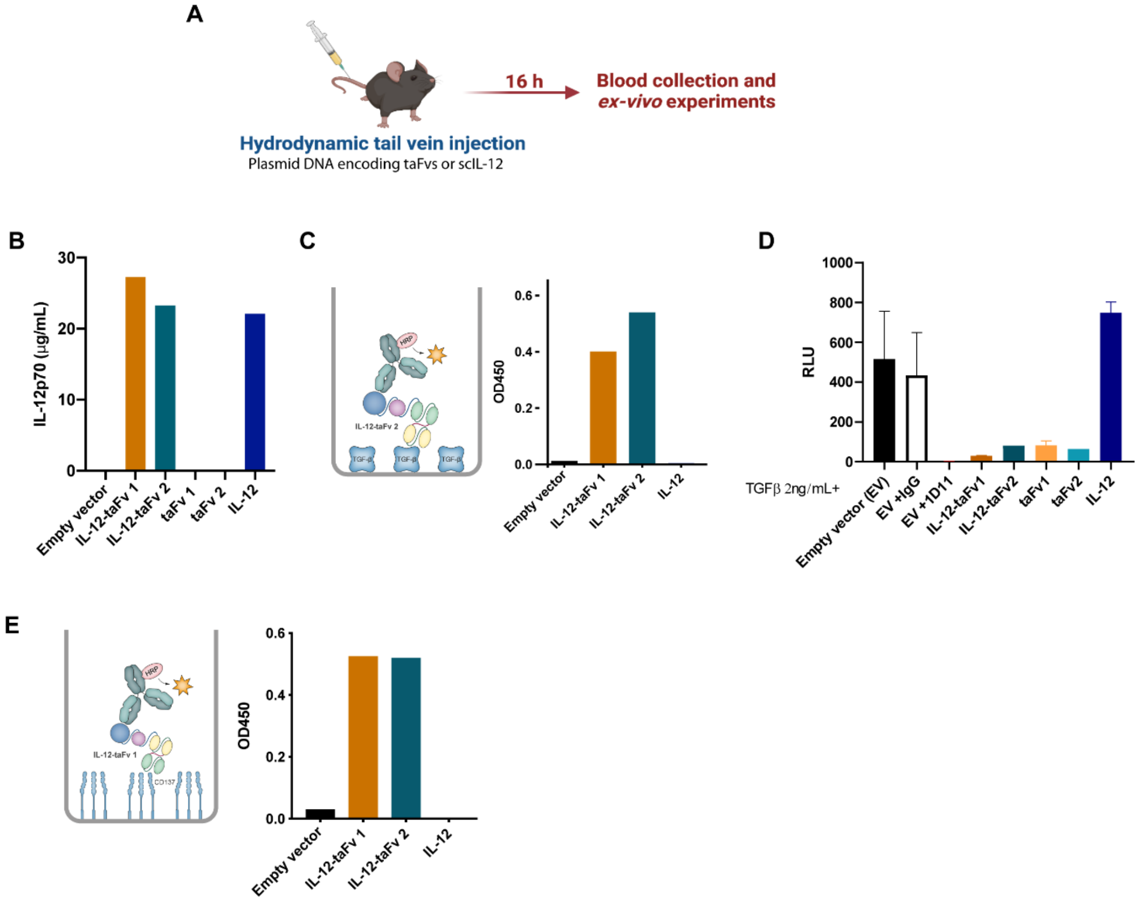

Figure S2

Figure S2. Hydrodynamic DNA-mediated gene transfer to the liver gives rise to circulating functional chimeric constructs.

(A) Schematic representation of the hydrodynamic gene transfer experiments and serum sample collection. (B) IL-12 concentration in the serum of the mice hydrodynamically gene-transferred with the indicated cDNA constructs. (C) Sandwich ELISA-type experiments, as in figure 1D performed on the serum samples. (D) Inhibition of anti-TGF-β bioactivity exerted by the sera of mice transferred with the indicated cDNA expression cassettes. Assays for TGF-β binding were as in figure 1E. (E) Binding of the constructs in the serum of mice to plastic absorbed-mCD137 in assays as those in figure 1F. Results are representative of two repetitions. In D, data are shown as mean±SEM.

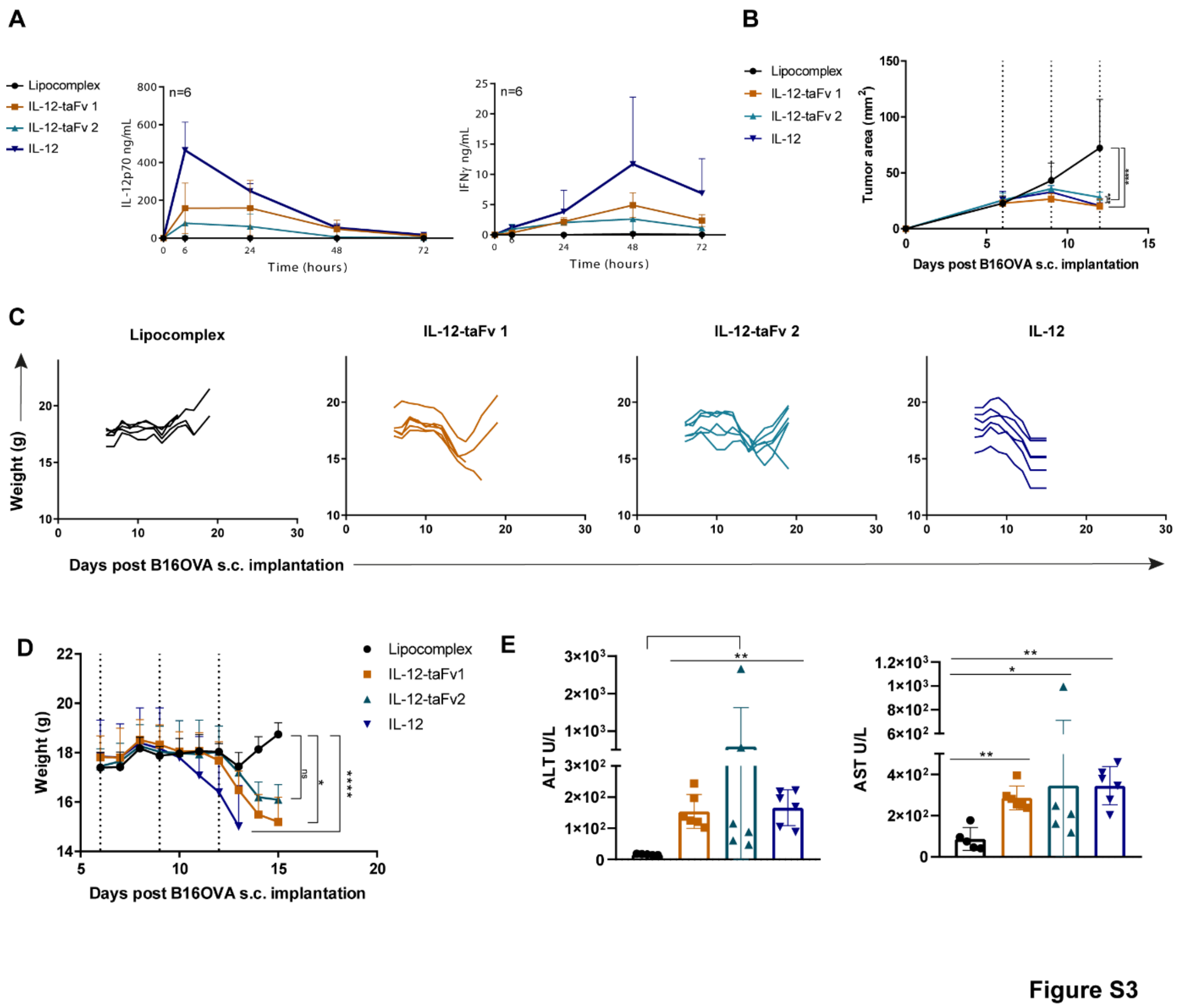

Figure S3. mRNA transfer to the liver gives rise to circulating functional levels of the chimeric constructs, exerts antitumor activity but results in toxic effects.

(A) Time course analyses of IL-12 and IFNγ concentrations in the blood of mice whose livers were transduced by 10 µg of lipoplexed mRNAs encoding the indicated constructs. (B) Tumor size follow-up of mice treated systemically with equimolar doses of the indicated mRNA constructs. Treatment doses were given when indicated by dotted lines. The experiment was finished when mice started to die due to toxicity. (C) Individual weight follow-up in mice from experimental groups in B. (D) Compiled data from C with statistical comparisons. (E) Transaminase concentrations in serum on day 13 from the mice treated as in B. Longitudinal data were fitted to a third-order polynomial equation and compared with an extra sum-of-squares F test (B and D). In E, the Mann-Whitney test was used for statistical comparisons. Data are expressed as mean ± SD. Statistical significance: *≤ 0.05, **p ≤ 0.01, ***p ≤ 0.001, ****p≤0.0001.

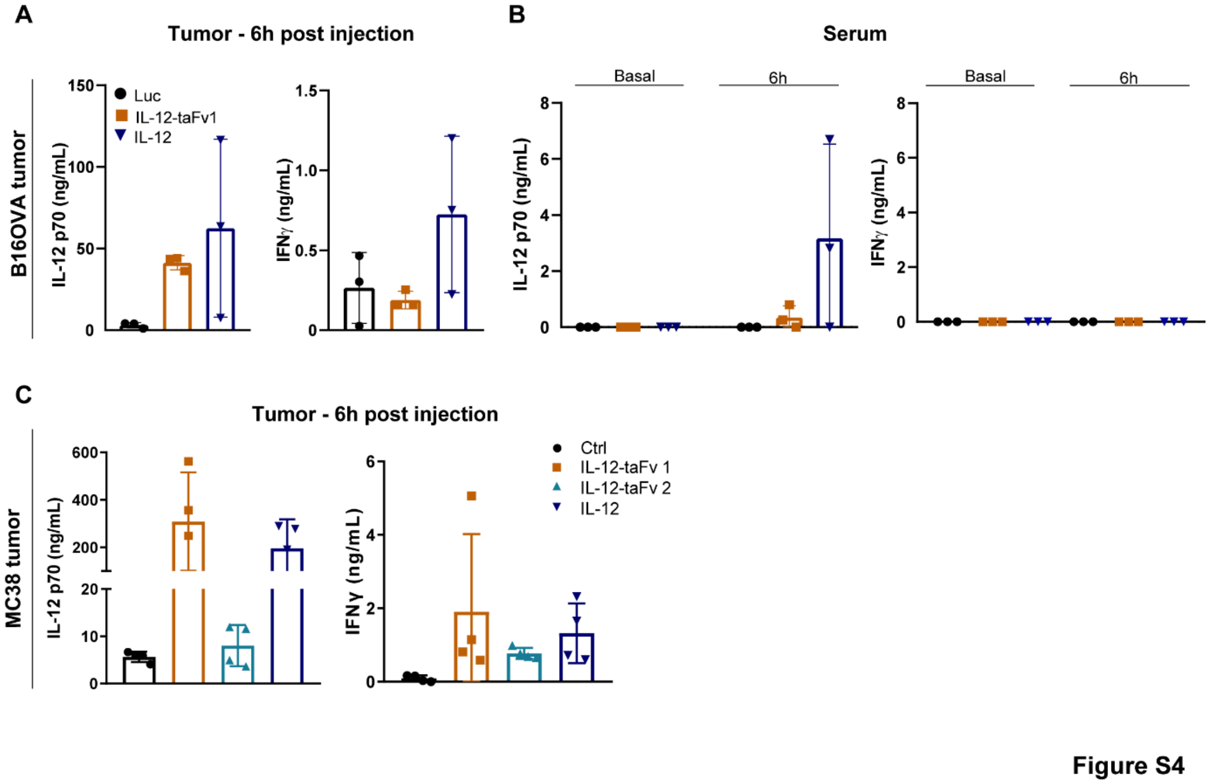

Figure S4. Intratumoral delivery of the chimeric mRNAs results in functional transgene expression in the tumor microenvironment.

(A) In mice bearing B16OVA treated as in figure 2, tumors were excised 6 hours post mRNA intratumoral injection of the indicated mRNAs. Tumor nodules were minced in a fixed volume of PBS with protease inhibitors and quantified by ELISA for IL-12 and IFNγ concentrations. (B) IL-12 and IFNγ concentrations in the serum samples from the mice are shown. (C) Similar experiments as in A with tumors derived from MC38 colon carcinoma cell line. Data are expressed as mean ± SD.

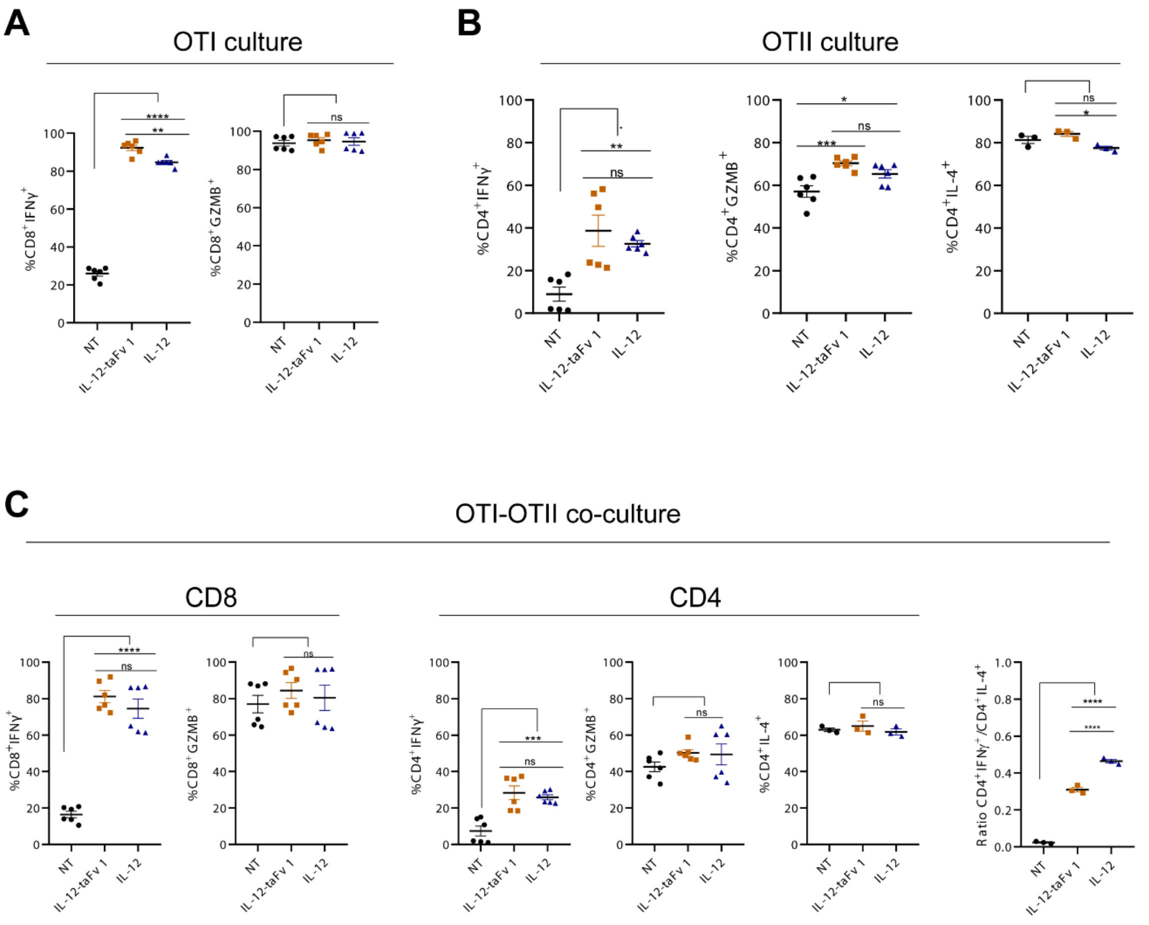

Figure S5

Figure S5. OTI and OTII lymphocytes undergoing antigen stimulation release more IFNγ upon exposure to the chimeric constructs. Splenocytes were isolated from OTI and OTII mice. Cells were activated with the respective OVA synthetic peptides and cultured in the presence of supernatants derived from 293T cells enriched for the indicated mRNA-encoded proteins. Following 48 hours, cells were stained and analyzed by flow cytometry. (A) represents OTI cultures, (B) represents OTII cultures, and (C) represents 1:1 mixture of OTI+OTII splenocytes. Results are representative of two repetitions with comparable outcomes. Statistical comparisons were made by one-way Anova followed by Tukey post-test. Data are expressed as mean± SEM. Statistical significance: *≤ 0.05, **p ≤ 0.01, ***p ≤ 0.001, ****p≤0.0001.

A

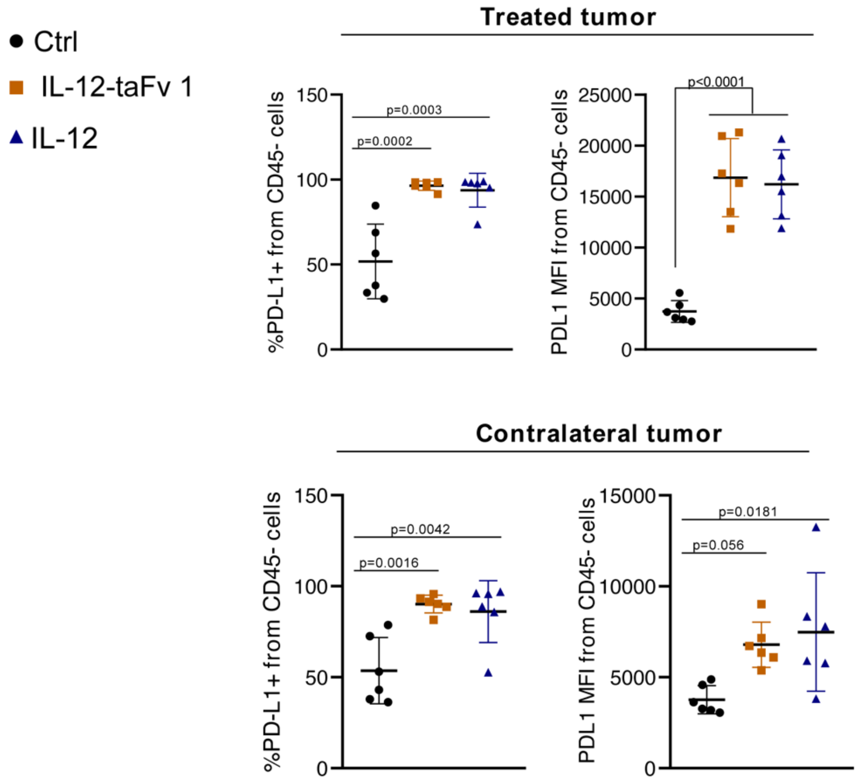

Figure S6

Figure S6. Intratumoral delivery of IL-12-containing chimeric mRNAs results in the upregulation of PD-L1 expression on tumor cells. B16OVA-bearing mice were intratumorally injected with 26.1 µmol of the indicated mRNAs in Ringer's lactate on day +8 and +9. 24h following the second injection, treated and distant non-injected tumors were excised and stained for flow-cytometry. Quantifications of percentages of $CD45^{-}PD\text{-}L1^{+}$ cells and PD-L1 MFI are shown. Results are representative of two repetitions with comparable outcomes. Statistical comparisons were made by one-way Anova followed by Sidak post-test. Data are expressed as mean± SD and p values are provided.
